# Access to Nature Has Always Been Important; With COVID-19, It Is Essential

**DOI:** 10.1177/1937586720949792

**Published:** 2020-08-17

**Authors:** A. Sachs Naomi

**Affiliations:** Co-editor

Did it have to take a pandemic for people to see the value of the outdoors? We may never know, but it is one of the silver linings in an atrocious situation. It is difficult at this time to think about anything without the lens of COVID-19. Not surprisingly, all three of our editorials for this issue of *HERD* address it. Another lens that I view almost everything through is nature and its potential to improve health and well-being. Getting healthcare administrators, providers, and designers to buy in to the value of the outdoors, and specifically access to nature in healthcare, has been a decades-long challenge. As I have written about in previous editorials, we had come to a point (pre-COVID) where access to nature in healthcare, through gardens and other outdoor spaces, was beginning to be viewed not just as “the icing on the cake” but as an important part of the overall environment of care.

***Did it have to take a pandemic for people to see the value of the outdoors?***

Since January, 2020, the world has been under the threat of a silent, invisible, highly contagious virus that is forcing individuals, communities, and society as a whole to rethink basic assumptions and best practices. Our relationship with the outdoors has changed. Most research indicates that the novel coronavirus is more easily transmitted indoors than outdoors (Qian et al., 2020; Samuel, 2020). Being outside is suddenly not just “nice,” it is potentially life-saving.

***Being outside is suddenly not just “nice,” it is potentially life-saving.***

## Are Healthcare Facilities Using Outdoor Space Differently?

I have been curious about whether outdoor spaces in healthcare facilities are being used differently due to the novel coronavirus, and if so, how. In March I posted a query about this on social media (LinkedIn, Facebook, and Twitter). I received a few replies, which I will share here.

A horticultural therapist with a large metropolitan healthcare organization reported that visitors were not using the gardens: “patients are allowed one visitor (same for every day) and when visitor leaves [after visiting the patient], they must leave the campus. So only employees in the gardens.” However, staff were using the gardens more than ever: “Staff are in private respite and trying to find coping space. Some walk. Some just breathe fresh air. Most seem to hover in the sun.”

***“Staff are in private respite and trying to find coping space. Some walk. Some just breathe fresh air. Most seem to hover in the sun.”***

Another horticultural therapist, from the same city but with a different healthcare organization, sent me an email:

Our garden groups have been very large since everyone is wanting to get outside rain or shine…The two therapeutic gardens…have also been invaluable during this time. I have held staff meetings, farewell gatherings, as well as annual reviews outside. One of our gardens is closed at this time for repair, so it has been nice for staff to take breaks in and walk laps. I have also talked about all the plants that are featured in the garden with staff, and gardening seems to be on the brain as a stress reducer.

An administrator for senior care facilities reported,

We have a much greater number of residents from both our retirement community and care homes using the gardens at the moment, along with their carers. Gardens have also been used as ‘stages’ to provide entertainment outside of residential buildings. More gardening activities are taking place, with balcony gardens and planters being put into use.

A landscape architect reported about a hospital that has four gardens,

The 4 beautiful Gardens have experienced a 3-4 times increase in the use of the walkways through the Gardens. The quantity of people is near the amount that show up when there is an event in the Gardens. Now is an open door to educate about the virtues of Nature in our lives.

## The Outdoors as Treatment Overflow

In some cities, large healthcare organizations have been converting the outdoor spaces of their urgent care and medical office building to spaces to treat people outdoors. Facilities from Washington state to Washington, DC have used or have plans to use their gardens and other outdoor spaces on their property, in tents or in the open air. The largest scale example in this country so far has been Mount Sinai Health System in New York City, which started treating patients in a 68-bed tent field hospital in Central Park in April (Fink, 2020).

***The largest scale example in this country so far has been Mount Sinai Health System in New York City, which started treating patients in a 68-bed tent field hospital in Central Park in April***

## Bringing Nature Indoors

The same healthcare system has also used tents in another way. At Mount Sinai Beth Israel, a former triage tent was converted into “recharge room” for stressed healthcare workers (Elliot, 2020). In early March, Dr. David Putrino, the director of rehabilitation innovation at Mount Sinai, asked the New York-based start-up Studio Elsewhere to design a space for hospital staff that would feel like an immersion in nature. The first space, boasting nature sounds and videos, live plants, and soft lighting, was built in a sub-basement of Mount Sinai on 98th Street. Ten more spaces at five Mount Sinai facilities have opened since. The triage tent recharge room at Beth Israel opened on May 20 (Elliot, 2020). In a survey of about 500 recharge room visitors, conducted by the hospital, researchers found that participants’ self-reported stress dropped 60% after just 15 min. As one doctor stated, “You go from hearing beeps and vents and whistles and all the intensity on the ward, with the bright lights, to this serene space. Suddenly something happens that really allows you to decompress almost immediately” (Elliot, 2020). Studio Elsewhere is continuing with their Frontline Strong Relief initiative, the goal being to “rapidly convert underutilized spaces in hospitals into relief hubs for workers to have rest and psychological reset during their day” (Studio Elsewhere, 2020).

## Nature for Health, and for Joy

In general (outside of healthcare) in the United States and internationally, people seem to be utilizing the outdoors more. Most countries and cities, even when enforcing strict stay-at-home orders, have allowed for and even encouraged citizens to go for short walks in their neighborhoods. Kaplan’s (1990) call for “nearby nature” has never been so resonant. Whether they are cycling on a trail rather than in the gym, eating outside the restaurant rather than inside, meeting a friend in a park rather than at a bar, or growing “victory gardens” for food rather than just flowers, many people are connecting with nature more than they have in years, if ever. Some of this is out of necessity. Gyms and bars are closed, and even if they are not, outside feels safer. Food grown at home means fewer trips to the grocery store, and perhaps some money saved. For parents, gardening is a way to engage their children and perhaps even teach some STEM in a creative and productive way.

The gardening app GrowIt reported a 10-fold growth in daily active users from March to May (White, 2020). ShrubBucket, an online plant seller, saw sales jump 13-fold over last year, and demand for vegetable plants jumped by almost 1,200% on a year-over-year basis (White, 2020). At the time of this writing, #covidgardening had 8,705 posts on Instagram. A survey by Civic Science found that 43% of Americans aged 13 and over said they would be engaging in more outdoor activities due to COVID-19 “social distancing” rules. Of those activities, hiking, visiting local parks, and boating/fishing were the most likely to see a boost (Brode, 2020).

In addition to feeling safer outdoors, people seem to be *enjoying* the outdoors more. They are discovering the beauty and joy that nature has to offer. As Atkinson (2020) of the University of Washington writes,

Americans have long turned to the soil in moments of upheaval to manage anxieties and imagine alternatives. My research has even led me to see gardening as a hidden landscape of desire for belonging and connection; for contact with nature; and for creative expression and improved health.

I have found the same in countless conversations (most via Zoom, email, and social media) and have experienced it personally. Our postage-stamp garden, which had been mostly paved before, is now filled with raised beds bursting with herbs, tomatoes, chard, and kale (and yes, I post pictures of all of these to #covidgardening on Instagram). Our daily neighborhood walks feel vital. The houseplants are beacons of hope and life. As one friend said, “It’s the only thing that feels normal.”

I hope very much that we are back to “normal,” whatever that is, before too long. That with rapid testing, and a vaccine, and perhaps even a cure, we can return to a semblance of life as we knew it before COVID-19. And I hope that when that happens, the appreciation for and value of nature, including in healthcare, remains.

Naomi A. Sachs, PhD, MLA, EDACCo-Editor, *Health Environments Research & Design Journal (HERD)*
